# Ultraviolet irradiation increases green fluorescence of dihydrorhodamine (DHR) 123: false‐positive results for reactive oxygen species generation

**DOI:** 10.1002/prp2.303

**Published:** 2017-03-10

**Authors:** Pascal Djiadeu, Dhia Azzouz, Meraj A. Khan, Lakshmi P. Kotra, Neil Sweezey, Nades Palaniyar

**Affiliations:** ^1^ Innate Immunity Research LaboratoryPhysiology and Experimental MedicinePeter Gilgan Centre for Research and LearningThe Hospital for Sick Children686 Bay StTorontoOntarioCanadaM5G 0A4; ^2^ Department of Pharmaceutical SciencesLeslie Dan Faculty of PharmacyUniversity of TorontoTorontoOntarioCanadaM5S 3M2; ^3^ Department of Laboratory Medicine and PathobiologyUniversity of TorontoTorontoOntarioCanadaM5G 1X8; ^4^ Department of PhysiologyUniversity of TorontoTorontoOntarioCanadaM5G 1X8; ^5^ Department of PediatricsUniversity of TorontoTorontoOntarioCanadaM5G 1X8; ^6^ Institute of Medical SciencesFaculty of MedicineUniversity of TorontoTorontoOntarioCanadaM5G 1X8

**Keywords:** Apoptosis, early and late apoptosis, extrinsic pathway Extra keywords if necessary Collectins, Fas‐Fas Ligand, lung inflammation, surfactant protein D (SP‐D), TNF‐TNFR

## Abstract

Dihydrorhodamine (DHR) 123 is a fluorophore commonly used for measuring reactive oxygen species (ROS), often after exposing cells to ultraviolet (UV) irradiation or oxidative burst inducers such as Phorbol 12‐myristate 13‐acetate (PMA). However, the negative effects of UV irradiation on oxidation of DHR123 itself to green fluorescence rhodamine (R) 123 under different experimental conditions (e.g., different buffers, media, cells, ROS detection techniques) have not been fully appreciated. We determined the effect of UV on DHR123 fluorescence, using a cell‐free system, and A549 epithelial cells, NIH/3T3 fibroblast cells, Jurkat T cells, primary human T cells, HL‐60 neutrophils and primary human neutrophils. We found that UV irradiation rapidly increases green fluorescence of DHR123 in cell‐free solutions. The intensity of green fluorescence increases with increasing amounts of DHR123 and UV exposure. The fluorescence increase was greater in Roswell Park Memorial Institute medium (RPMI) than DMEM media. The presence of DMSO (0–1.25%, v/v) in RPMI further increases the fluorescence signal. Phosphate buffered solution (PBS) and Hanks' Balanced Salt Solution (HBSS) generate considerable background signal with DHR123, and increasing DMSO concentration greatly increases the fluorescence signal in these buffers. However, after UV irradiation the amount of DHR123 that remains unoxidized generates sufficient fluorescence signal to measure the ROS produced by H_2_O_2_ and peroxidase in vitro or Nicotinamide adenine dinucleotide phosphate (NADPH) oxidase‐mediated ROS production within HL‐60 neutrophils or primary human neutrophils. We conclude that UV irradiation oxidizes DHR123 to generate Rhodamine 123 (R123) green fluorescence signal, and that the R123 present in the culture supernatant could give erroneous results in plate reader assays. However, flow cytometry and fluorescence microscopy reliably detect ROS in cells such as neutrophils. Overall, avoiding false‐positive results when detecting ROS using DHR123 requires selection of, agonists, the correct buffers, media, cell types, and measurement techniques.

**Abbreviations**
ANOVA: Analysis of variance
DAPI: 4′, 6‐diamidino‐2‐phenylindole
DHR123: Dihydrorhodamine 123
DIC: Differential interference contrast
DMEM: Dulbecco's Modified Eagle Medium
DMSO: Dimethyl Sulfoxide
FI: Fluorescence Intensity
H_2_O_2_: Hydrogen peroxide
HBSS: Hanks' Balanced Salt Solution
MFI: Median fluorescence intensity
NADPH: Nicotinamide adenine dinucleotide phosphate
NOX: NADPH oxidases
PBS: Phosphate buffered solution
PMA: Phorbol 12‐myristate 13‐acetate
R123: Rhodamine 123
ROS: Reactive oxygen species
RPMI: Roswell Park Memorial Institute medium

## Introduction

Dihydrorhodamine 123 (DHR123) is a fluorescence probe commonly used for measuring reactive oxygen species (ROS) (Crow 1997). Several types of cells produce ROS or reactive nitrogen species during oxidative stress, cell death, exposure to UV irradiation or agonists such as PMA in the form of superoxide, peroxynitrite, H_2_O_2_, and singlet oxygen molecules (Crow 1997; Kalyanaraman et al. 2012). In the presence of ROS, nonfluorescent DHR123 is oxidized to rhodamine (R123). When R123 is excited at 488 nm, it emits a green fluorescence signal with peak intensity at 525 nm (Qian and Buettner 1999). Therefore, ROS production is routinely measured with DHR123 using fluorescence plate reader assays, flow cytometry or fluorescence microscopy. DHR123 has been used for measuring ROS both in cell‐free conditions (Shen et al. 2014) and in cell‐based systems with human neutrophils, neutrophils differentiated from HL‐60 cell line (Chen and Junger 2012; Douda et al. 2014, 2015), eosinophils (Elsner et al. 1996), lymphocytes (Poniedzialek et al. 2015), epithelial cells (Wilhelm et al. 2009; Kelley et al. 2013) and mixed cell cultures (Hanson and Clegg 2002). However, some of these cell types do not have the peroxidases needed to convert H_2_O_2_ to HOCl, a substrate necessary to effectively convert DHR123 to fluorescence R123 (Furtmuller et al. 2000). Therefore, using DHR123 in all of these cells to measure UV‐ or PMA‐mediated ROS production requires careful re‐evaluation.

In this study, we aimed to analyze the direct effect of UV irradiation on DHR123 dissolved in commonly used buffers and media, and in four different cell types: T cells, fibroblasts, epithelial cells, and neutrophils. We found that UV irradiation alone increases the green fluorescence signal of DHR123 and thus could lead to erroneous conclusions in plate reader based assays. However, the oxidative effect of UV light on DHR123 does not interfere with flow cytometry‐ and fluorescent microscopy‐based analysis (e.g., neutrophils). DHR123 is appropriate for measuring PMA‐mediated ROS production in neutrophils, but not in epithelial, fibroblast and T cells. Overall, great care should be taken in the selection of buffers, media, and cell types when using DHR123 to measure ROS production. It is particularly important because background buffers and cells are not UV irradiated with DHR123. Therefore, correcting buffer or cell background would not correct the background effect of UV on DHR123‐mediated increase in fluorescence in these assays.

## Materials and Methods

### Reagents and buffers

Dulbecco's Modified Eagle's Medium (DMEM) and Roswell Park Memorial Institute medium (RPMI) were purchased from Wisent Inc. (Quebec, Canada). Hanks' Balanced Salt Solution (HSBB) and Phosphate Buffered Solution (PBS) were purchased from Life Technologies (California, United States). DHR123 and Dimethyl Sulfoxide (DMSO) were purchased from Sigma‐Aldrich (Missouri, United States). Hydrogen peroxide (H_2_O_2_) was purchased from Thermo Fisher Scientific (Massachusetts, United States).

### Cell culture

Jurkat T cells (an immortalized line of human T lymphocytes) were cultured in RPMI. A549 cells (a human lung epithelial cells line, ATCC), mouse NIH/3T3 fibroblast cells (a fibroblast from mouse embryo, ATCC) and HL‐60 cells (a human promyelocytic leukemia cell line, ATCC) were cultured in DMEM. All media were supplemented with 10% (v/v) fetal bovine serum (Invitrogen), and the cells were grown at 37°C in the presence of 5% CO_2_ (v/v). A concentration of 1 × 10^6^ cells per mL was maintained throughout the experiments.

### Human primary T cell and neutrophils isolation

Peripheral blood from healthy donors was obtained with informed consent, and the study was approved by the Research Ethics Board of the Hospital for Sick Children. Peripheral blood mononuclear cells (PBMCs) were isolated by gradient centrifugation in Lymphoprep (Axis‐Shiel, Oslo, Norway) following the manufacturer's instructions. The isolation of human T cells was performed using the Human T‐cell Enrichment Kit (STEMCELL Technologies Inc, BC, Canada). Isolated T cells were re‐suspended in RPMI supplemented with 10 mmol/L HEPES for UV irradiation experiments.

Human peripheral neutrophils were isolated from blood collected in K2 EDTA blood collection tubes (Becton, Dickinson and Co.) using PolymorphPrep (Axis‐Shield) according to the manufacturer's recommendation. Isolated neutrophils were then re‐suspended in RPMI medium (Invitrogen) supplemented with Hepes buffer (10 mmol/L, pH 7.2) for UV irradiation and PMA‐mediated ROS production experiments.

### UV irradiation

BD 96‐well plates were used for irradiation experiments and irradiation with UV light was performed using the Stratagene UV Stratalinker 2400 which emits UV light at 254 nm wavelengths and delivers 75 J/sec.

### Fluorescence plate reader assays

The Spectra Max Gemini EM plate reader (Molecular Devices) was used for fluorescence measurement. To detect and quantify ROS production from cells, 25 *μ*mol/L DHR123 was used. DHR123 was either incubated with the buffer alone or with the cells at 37°C for 15 min, followed by three washes at 400g for 5 min. The Spectra Max Gemini EM plate reader settings, 488 nm for excitation and 525 nm for emission, were used for measuring the R123 green fluorescence signal, unless otherwise stated. BD Falcon 96‐well plates and 100 *μ*L of solution per well were used in all experiments.

### Evaluation of the effect of increased irradiation with UV light on DHR signal

DHR123 (different concentrations, 0–20 *μ*mol/L) in 100 *μ*L HSBB was placed in the wells of a 96‐well plate. The wells were irradiated with UV light for various periods of time ranging from 0 to 5 min. Fluorescence intensity was measured using the Spectra Max Gemini EM plate reader.

### Evaluation of the effect of media, buffer and DMSO concentration on DHR signal

Solutions with different concentrations of DMSO (0–1.25%, v/v) with and without 25 *μ*mol/L DHR123 were prepared in two media (RPMI and DMEM) and two buffers (PBS and HSBB). The volume of 100 *μ*L of each buffer solution was loaded onto 96‐well plates and irradiated for 30 sec with UV light. Fluorescence intensity was measured using the Spectra Max Gemini EM plate reader.

### Measuring ROS production in cells incubated with DHR123 before UV irradiation

A549 epithelial cells, 3T3 fibroblast cells, Jurkat T cells, primary T cells, HL‐60 differentiated to neutrophils and primary neutrophils (10^6^ cells/mL) were incubated with 25 *μ*mol/L DHR123 for 15 min. Cells were then centrifuged twice at 400*g* for 5 min and re‐suspended in fresh RPMI media. A volume of 100 *μ*L of cells suspension was plated in a well within a 96‐well plate (10^5^ cells per well). The cells were treated with and without PMA and irradiated for 30 sec with UV light. Oxidized R123 green fluorescence signal was measured using the Spectra Max Gemini EM plate reader.

### Measuring ROS production in cells incubated in DHR123 after UV irradiation

A549 epithelial cells, 3T3 fibroblast cells, Jurkat T cells, primary T cells, HL‐60 differentiated to neutrophils and primary neutrophils (10^6^ cells/mL) were plated onto BD Flacon 24‐well plates (10^6^ cells per well) and irradiated for 30 sec with UV light. The cells were incubated for 15 min with 25 *μ*mol/L DHR123. The cells were then centrifuged at 400*g* for 5 min and resuspended in fresh RPMI media. The procedure was repeated twice. The cells were plated onto 96‐well plates (10^5^ cells per well). ROS signal was measured using the Spectra Max Gemini EM plate reader.

### Amount of DHR remaining after irradiation with UV light

Solutions with various concentrations of H_2_O_2_, 1:10,000 donkey‐anti rabbit HRP‐conjugated antibody as a source of peroxidases and 25 *μ*mol/L DHR123 were prepared in HSBB. A volume of 100 *μ*L solutions was loaded onto 96‐well plates and irradiated for 30 sec with UV light. Fluorescence intensity was measured using the Spectra Max Gemini EM plate reader.

### Flow Cytometry

To evaluate ROS production in Jurkat T cells and HL‐60 cells, cells were suspended in RPMI media supplemented with 10 mmol/L HEPES. Cells were further treated with 25 *μ*mol/L DHR123 by incubation at 37°C for 15 min followed by three washes. One batch of cells was treated with 25 nmol/L PMA and the other batch of cells was irradiated for 30 sec. Irradiated, PMA‐treated and control cells without irradiation or PMA treatment were cultured over 1 h, and ROS production was analyzed by counting 10^4^ events using the 488 filter and the FITC laser of the Gallios (Beckman Coultler Inc) flow cytometer.

### Fluorescence Microscopy

One‐half of the cells used for flow cytometry were used for fluorescence microscopy, and the experiment (UV or PMA treatment) was repeated as above. After 1‐h incubation of 10^5^ cells/well in 96‐wells plate (black plate with clear bottom, BD) were fixed with 1% (v/v) PFA for 15 min at 4°C. Cells were then washed three times with PBS and visualized using the Nikon TE‐2000 epifluorescence microscope (Nikon). Images were further analyzed using the Volocity software (Perkin Elmer).

### Statistical analysis

Mean, variances, standard errors, and statistical significance (*P *<* *0.05) values were calculated by the analysis of variance or ANOVA, Dunnett's test or student *t*‐test as appropriate using Prism software (Prism Software Corp.). Data are reported as mean ± Standard Error of Mean (SEM).

## Results

### Irradiating DHR123 with UV light generates green fluorescence

**Figure 1:**
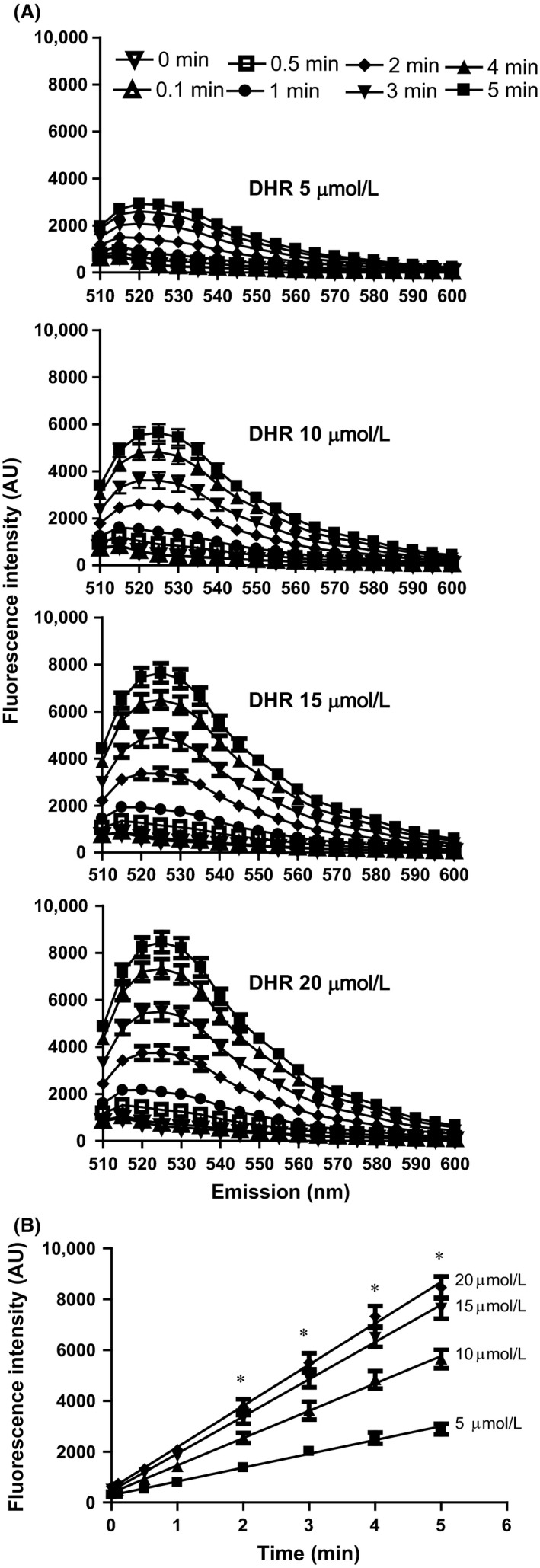
UV irradiation increases green fluorescence signal of DHR123. (A) The effect of UV irradiation time on green florescence emission from DHR123 solutions was analyzed. Various concentrations of DHR123 (5–20 *μ*mol/L) diluted in RPMI were irradiated with UV for various time periods (0–5 min). With a fixed excitation at 488 nm, emission was measured between 510 nm and 600 nm. (B) Linear regression of the fluorescence intensity on the irradiation period shows a probe concentration‐ and irradiation duration‐dependent effect on DHR123 fluorescence. Regression lines: for 20 *μ*mol/L, *y* = 1620 x + 565, *r* ^*2* ^= 0.99; for 15 *μ*mol/L, y = 1463x + 460, *r* ^*2* ^= 0.99; for 10 *μ*mol/L, y = 1073 x + 386, *r* ^*2* ^= 0.99; for 5 *μ*mol/L, y = 543 x + 286, *r* ^*2* ^= 0.99). Data were analyzed by two‐way ANOVA with Bonferroni post‐tests. *P <* *0.05 indicates that values at each time point are significantly different from each other. *N* = 3; Experiments were conducted on three different days; each sample was analyzed in duplicate wells. RMPI, Roswell Park Memorial Institute medium.

In order to determine the direct effect of UV irradiation on DHR123, we measured the intensity of the peak fluorescence in the green range of the spectrum (525 nm) that was generated after irradiating various concentrations of DHR123 with UV light for different time periods. At each DHR123 concentration (5–20 *μ*mol/L), exposure of the probe to UV light for increasing durations (0–5 min) increased the green fluorescence signal in direct proportion to the duration of exposure to UV light (Fig. 1A). The slopes of these graphs increased with increasing DHR123 concentration, showing that more DHR123 is oxidized at higher concentrations (Fig. 1B).

**Figure 2:**
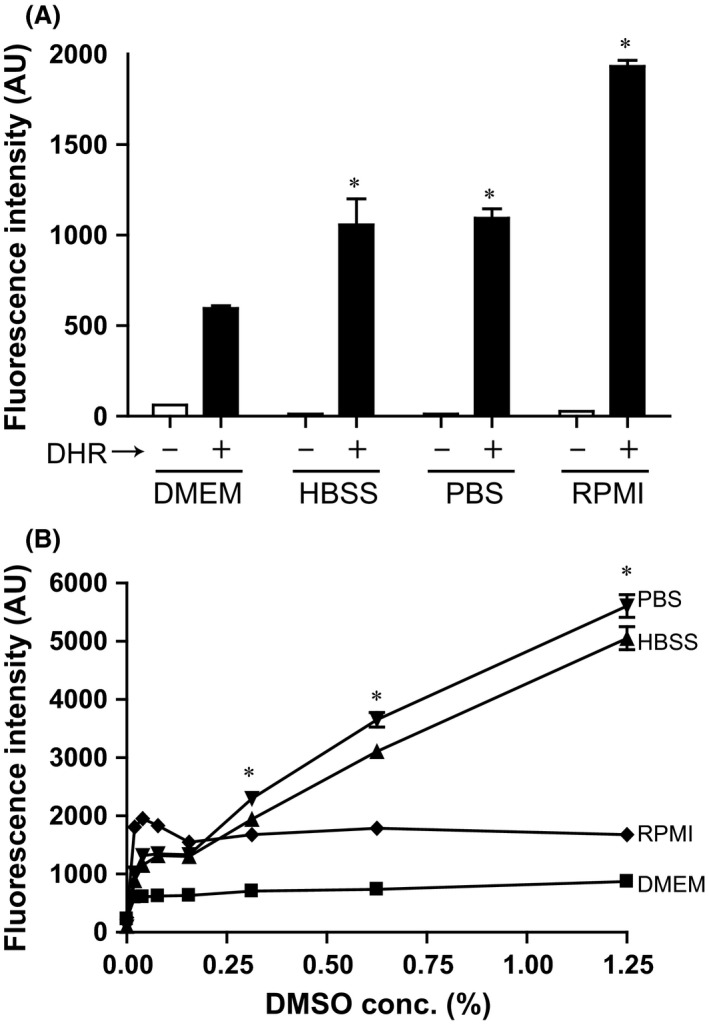
The UV‐mediated increase in green fluorescence emission of DHR123 varies in a buffer‐ and DMSO‐dependent manner. (A) UV irradiating 25 *μ*mol/L of DHR123 in different buffers shows the effect of RPMI, DMEM, HBSS, and PBS on oxidized DHR123 fluorescence intensity after UV irradiation. *indicates that values are significantly different than that of DMEM. Note that for every buffer or media condition, filled bar (with DHR123) is also significantly different than open bars (no DHR)(*P *<* *0.05). *N* = 3. B, Use of various DMSO concentrations (0–1.25%, v/v) in the presence of DHR123 and irradiation with UV for 30 sec show the direct effect of DMSO on UV‐induced green fluorescence signal generation in different buffers. *indicates that values at each DMSO concentration are significantly different from each other – PBS, HBSS, RPMI, DMEM (*P *<* *0.05). *N* = 3; Experiments were conducted in 3 different days; each sample was analyzed in duplicate wells. Data were analyzed by two‐way ANOVA with Bonferroni post‐tests. DMEM, Dulbecco's Modified Eagle Medium; DMSO, Dimethyl Sulfoxide; HBSS, Hanks' Balanced Salt Solution; RMPI, Roswell Park Memorial Institute medium.

To determine the effect of a variety of buffers and media on UV‐induced oxidative effect on DHR123, a concentration of 25 *μ*mol/L DHR123 was diluted in RPMI, DMEM, HBSS and PBS, and the solutions were irradiated with UV for 30 sec. Green fluorescence (525 nm) measurements showed higher fluorescence intensity for RPMI medium, followed by HBSS and PBS buffers (*P* < 0.05). DMEM media showed the lowest fluorescence response after irradiating DHR123 with UV light (Fig. 2A).

DMSO is often used as a solvent for DHR123 and other compounds. To determine the effect of DMSO on the UV‐induced increase in green fluorescence signal of DHR123, experiments were repeated with various concentrations of DMSO in these four buffers and media. The presence of DMSO in DMEM increased the amount of green fluorescence; however, further increases in DMSO concentration did not affect the green fluorescence signal. Furthermore, the presence of DMSO at any of the tested concentrations (0–1.25%, v/v) had little effect on DHR fluorescence signal in RPMI media. By contrast, increasing concentrations of DMSO steadily increased the green fluorescence signal of DHR123 in PBS and HBSS buffers (Fig. 2B)**.** Therefore, concentration of dye, duration of UV irradiation, types of media, and types of buffers all differentially affect DHR123 green fluorescence signal. This point is important because during experiments conducted with cells, the background is typically subtracted using buffers and cells with no UV irradiation. Since these control buffers and cells do not emit green fluorescence without UV irradiation, any UV‐induced DHR123 background signal will not be corrected.

**Figure 3:**
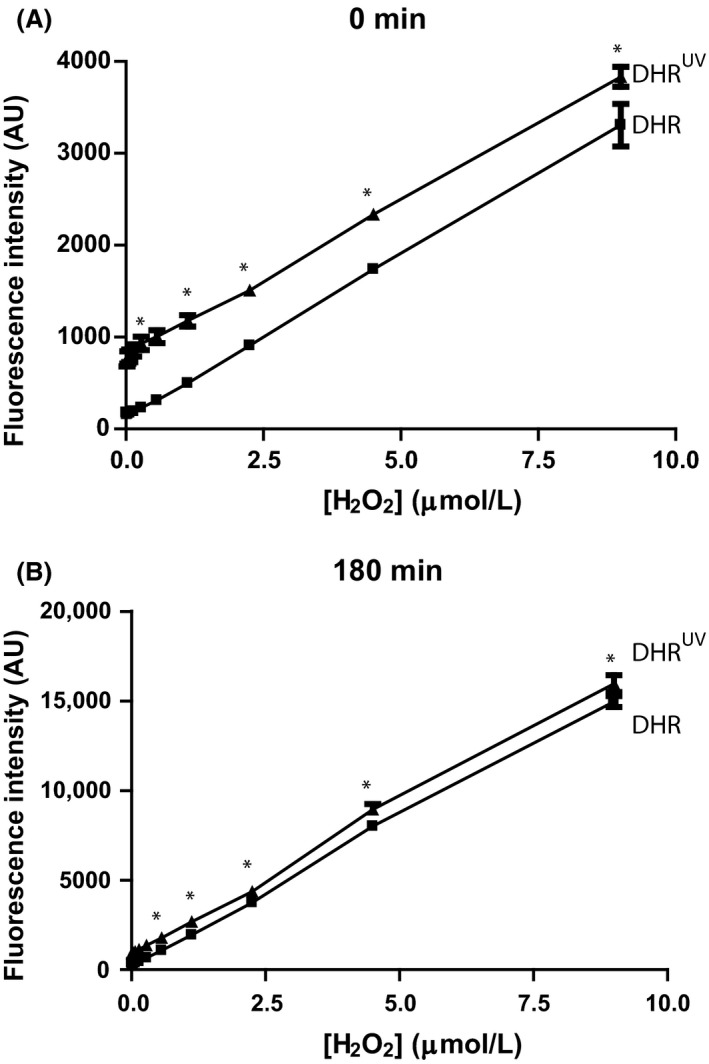
Substantial amount of unoxidized DHR123 is available to react with reactive oxygen species, after typical UV irradiation experiments. (A) DHR123 (25 *μ*mol/L) was irradiated and incubated in the presence of various concentrations of H_2_O_2_ and HRP, and the oxidation of DHR123 was measured at time 0 compared to control DHR123 with no irradiation. The effect of different H_2_O_2_ concentrations was analyzed by comparing the fluorescence intensity. *indicates that values for DHR123^UV^ (DHR^UV^) versus DHR123 (DHR) at each concentration is significantly different from each other. DHR^UV^ denotes that the DHR123 used in these samples was irradiated with UV light before the experiment. B, Green fluorescence signal detected in samples with different concentrations of H_2_O_2_ analyzed 3‐h post incubation show the effect of time on H_2_O_2_ peroxidase‐mediated DHR123 oxidation. *N* = 3; Experiments were conducted in 3 different days; each sample was analyzed in duplicate wells. Data were analyzed by two‐way ANOVA with Bonferroni post‐tests (*P* < 0.05).

To determine whether UV irradiation depletes the DHR123 available to react with ROS, we conducted a solution‐based assay. The DHR123 was irradiated with UV as shown above (Fig. 1) and further oxidized with H_2_O_2_ in the presence of peroxidase enzyme. UV irradiated DHR123 increased green signal compared to nonirradiated DHR123 at the 0 time point. In both of these samples (UV irradiated or non‐irradiated DHR123), green fluorescence signal increased over time. Compared to the 0 time point, the signal increased by a factor of >3.75 at the 3‐h time point (Fig. 3A, B). Thus, there are large amounts of unoxidized DHR123 available to react with ROS even after irradiating the dye with UV. Furthermore, at the later time points, the differences between the UV‐irradiated and non‐irradiated samples became small, suggesting that when large amounts of ROS are produced, the presence of pre‐oxidized DHR123 has little effect on the ability to detect ROS by the remaining DHR123.

### Rapid increase in green fluorescence in DHR123 is attributable to UV irradiation

**Figure 4:**
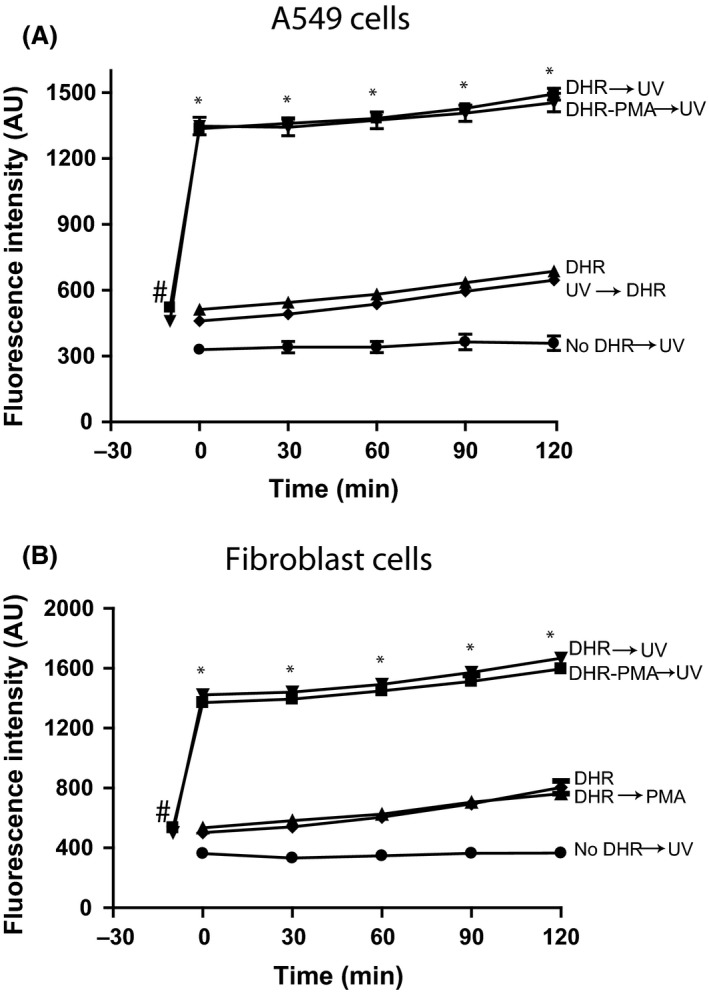
UV irradiation of DHR123‐preloaded epithelial and fibroblast cells results in a rapid increase in green fluorescence signal. (A) DHR123 (25 *μ*mol/L) was incubated with A549 lung epithelial cells in RPMI and irradiated for 30 sec. For another set of cells, DHR123 was added to the irradiated A549 cells and incubated at 37°C for up to 2 h. The incubation of DHR123 with A549 cells with no irradiation shows a background fluorescence compared to A549 cells with DHR123 added after irradiation of the cells. * indicates that fluorescence values for DHR123 followed by UV irradiation (DHR→UV) condition at each time point are significantly different from all the other conditions, except with (DHR‐PMA→UV). Fluorescence values for DHR versus DHR→UV or No DHR versus No DHR→UV are not significantly different from each other at any time point. ^#^indicates that the DHR→UV value is significantly different from No DHR→UV value. (B) Fibroblast 3T3 cells were also loaded with DHR123 (25 *μ*mol/L), similar to that of A549 cells. The results were also similar to that of A545 cell experiments. The use of PMA and UV does not show any difference compared to UV alone. *N* = 4; Experiments were conducted in four different days; each sample was analyzed in duplicate wells. Data were analyzed by two‐way ANOVA with Bonferroni post‐tests (*P *<* *0.05). RMPI, Roswell Park Memorial Institute medium.

**Figure 5:**
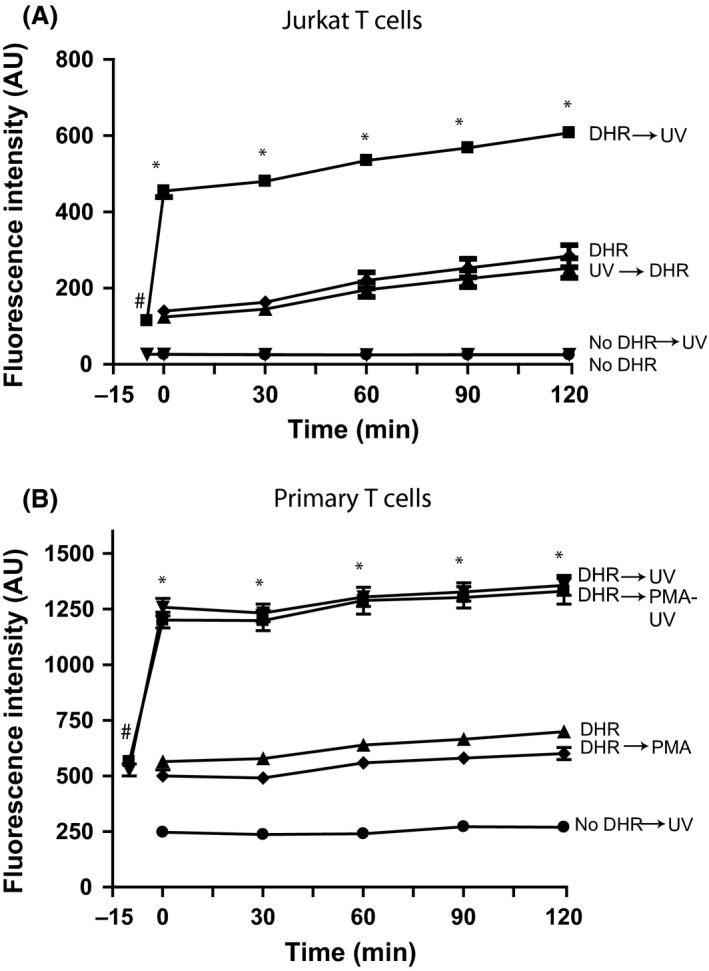
UV irradiation, but not the ROS produced by T cells, rapidly increases green fluorescence of DHR123. (A) DHR123 (25 *μ*mol/L) was incubated with Jurkat T cells in RPMI and irradiated for 30 sec. For another set of cells, DHR123 was added to the irradiated Jurkat T cells and incubated at 37°C for up to 2 h. The incubation of DHR123 with Jurkat T cells with no irradiation shows a background fluorescence compared to Jurkat T cells with DHR123 added after irradiation of the cells. *indicates that fluorescence values for DHR123 followed by UV irradiation (DHR→UV) condition at each time point are significantly different from all the other conditions. ^#^ indicates that the DHR→UV value is significantly different from No DHR→UV value. Fluorescence values for DHR versus DHR→UV or No DHR versus No DHR→UV are not significantly different from each other at any time point. *N* = 3; Experiments were conducted in three different days; each sample was analyzed in duplicate wells. (B) Primary T cells were also treated with DHR123 (25 *μ*mol/L), similar to that of Jurkat T cells. The results were also similar to Jurkat cell experiments. *indicates that fluorescence values for DHR123 followed by UV irradiation (DHR→UV) condition at each time point are significantly different from all the other conditions, except with (DHR‐PMA→UV). ^#^ indicates that the DHR→UV value is significantly different from No DHR→UV value. The use of PMA and UV does not show any difference compared to UV alone. *N* = 3; 3 different donors; each sample was analyzed in duplicate wells. Experiments were conducted in three different days. Data were analyzed by two‐way ANOVA with Bonferroni post‐tests (*P *<* *0.05). ROS, reactive oxygen species; RMPI, Roswell Park Memorial Institute medium.

DHR123 is often used for measuring ROS production in various cells. Therefore, to determine whether UV irradiation affects DHR123 signal in the context of cell‐based experiments, we used A549 epithelial cells (Fig. 4A), 3T3 fibroblasts (Fig. 4B), Jurkat T cells (Fig. 5A), and primary T cells (Fig. 5B). These cells were cultured in DMEM or RPMI, incubated with or without DHR123, washed and re‐suspended in RPMI, irradiated with UV light, and the green fluorescence signal was measured. DHR123‐loaded cells showed higher fluorescence signal compared to cells with no DHR123 at the beginning of the experiment (e.g., −5 or 0 min time points; Figs. 4, 5). This reading is consistent with the presence of some intrinsic R123 background green fluorescence of the DHR123 probe. A gradual increase in background green fluorescence was observed over the experimental period. No notable signal change was observed in the cells that were irradiated in the absence of DHR123. By contrast, the fluorescence signal of the DHR123‐loaded cells rapidly increased immediately after UV irradiation (Figs. 4, 5). Adding DHR123 to the cells immediately after UV irradiation did not show any rapid increase in green fluorescence signal. Collectively, these data indicate that UV irradiating A549 cells, 3T3 fibroblasts, Jurkat and primary T cells did not generate ROS detectable by DHR123, instead UV irradiating the cells that contained DHR123 showed a spike in increased green signal. This initial signal increase is attributable to the direct effect of UV light on DHR123. Therefore, this assay gives false positive results when UV irradiating DHR123‐loaded epithelial, fibroblast and T cells.

### DHR123 is good at detecting large amounts of ROS in neutrophils

**Figure 6:**
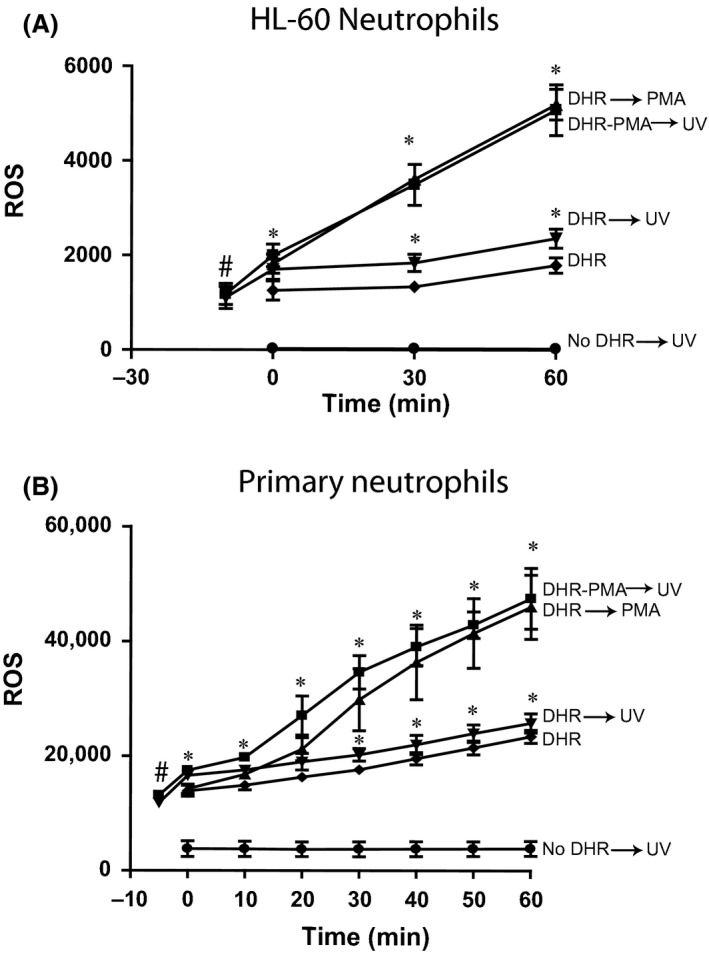
When the ROS production is large within cells, DHR123 is usable with or without UV irradiation in neutrophils. (A) HL‐60 neutrophils were incubated with 25 *μ*mol/L DHR123 at 37°C for 15 min. Cells were washed three times (400*g* for 10 min). Cells were either irradiated with UV for 30 sec or treated with 25 nmol/L PMA or both and incubated at 37°C in 96‐well plate for 2 h. Plate was read at 525 nm to measure oxidized DHR123 to determine ROS production. *fluorescence intensity shows the presence of a large amount of ROS in HL‐60 neutrophils treated with PMA compared to untreated cells or cells irradiated with UV (*P *<* *0.05). DHR versus DHR→UV values are significantly different from each other at each time point. *N* = 3; 3 different days; each sample was analyzed in duplicate wells. (B) Human primary neutrophils were treated as of the protocol described in A. The results were similar to that of HL‐60 neutrophils; however, the ROS values were relatively higher in primary neutrophils. *N* = 3; 3 different donors; each sample was analyzed in duplicate wells. Experiments were conducted in three different days. Data were analyzed by two‐way ANOVA with Bonferroni post‐tests. ROS, reactive oxygen species.

Neutrophils are known to generate large amounts of ROS after activating their NADPH oxidase with PMA. Therefore, to determine the effect of UV irradiation and NADPH oxidase‐dependent activation of neutrophils, we differentiated promyelocytic HL‐60 cells into neutrophils (Douda et al. 2015). Differentiated HL‐60 (Fig. 6A) and primary neutrophils freshly isolated from blood (Fig. 6B) were loaded with DHR123 and either irradiated with UV or incubated with PMA or both. Monitoring green fluorescence signal showed that neutrophils, but not Jurkat T cells (Fig. 5), generated large amounts of ROS that are detectable by DHR123 (Fig. 6). Similar to Jurkat and primary T cells, UV irradiation of DHR123‐loaded neutrophils also showed the initial spike in green fluorescence (note the scale difference between Figs. 5, 6). However, generation of a large amount of ROS still allows the detection of PMA‐induced NADPH oxidase‐mediated production of ROS by this method.

### Flow cytometry and fluorescence microscopy are more suitable for detecting ROS in cells compared to plate reader assays

**Figure 7:**
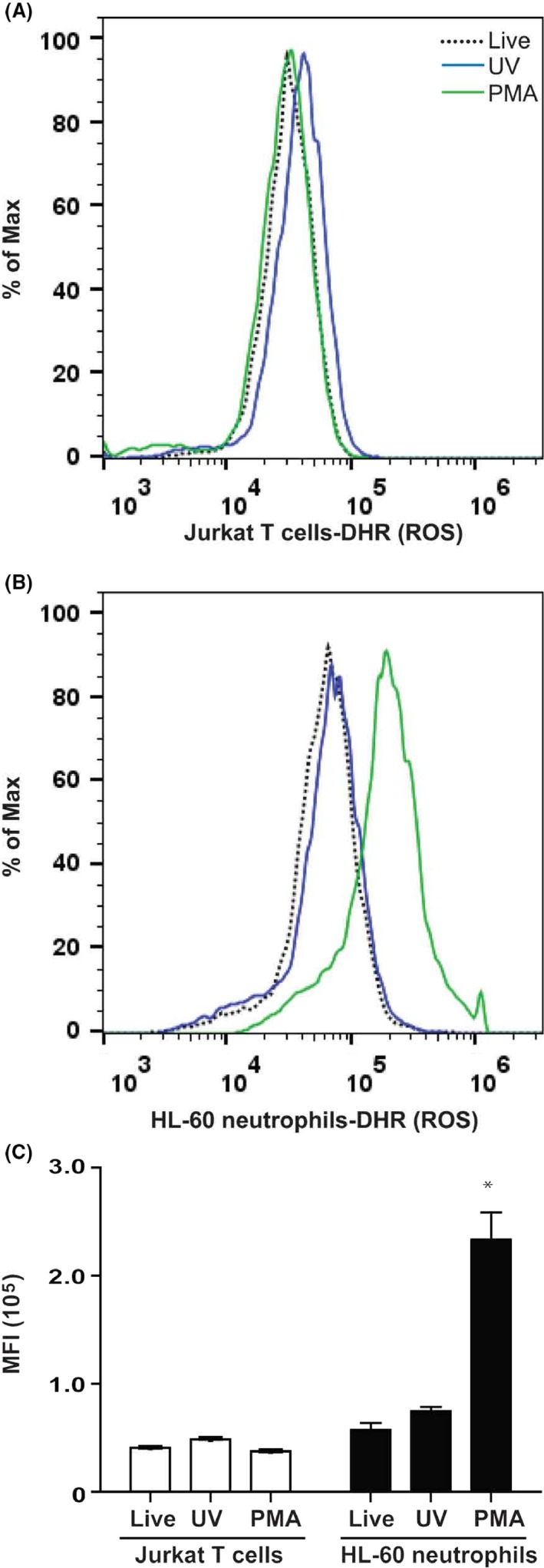
Flow cytometry correctly detects the presence of ROS inside the cells. Jurkat T cells and HL‐60 neutrophils were treated with 25 nmol/L PMA or irradiated with UV light. Fluorescence intensity was measure after 1‐h post incubation with DHR123. (A) Flow cytometry detects no ROS signal in Jurkat T cells after 60 min. (B) Flow cytometry detects a large amount of ROS in >75% of HL‐60 neutrophils compared to live cells or UV irradiated cells. (C) Mean fluorescence intensity shows that HL‐60 neutrophils treated with PMA generate large amounts of ROS compared to T cells and all other conditions (*,*P* < 0.05). *N* = 3; Experiments were conducted in three different days; each sample was analyzed in duplicate wells. Representative tracings are shown in A and B. Data were analyzed by two‐way ANOVA with Bonferroni post‐tests. ROS, reactive oxygen species.

The presence of extra DHR123 in the culture solutions or inside the cells during UV irradiation or PMA treatment could give erroneous results in plate‐reader assays. To evaluate whether flow cytometry and fluorescence microscopy are better methods, we conducted experiments with Jurkat T cells and neutrophils differentiated from HL‐60 cells. These cells were first incubated with 25 *μ*mol/L DHR123, washed, and then either treated with PMA or exposed to UV light. Cells were subsequently analyzed by flow cytometry after incubating them at 37°C for 1‐h. UV‐irradiated Jurkat T cells showed no significant difference in fluorescence intensity compared to non‐irradiated or PMA treated cells (Fig. 7A). By contrast, HL‐60 neutrophils showed a high level of ROS production that is readily measurable by DHR123 when treated with PMA, compared to non‐treated cells or cells irradiated with UV (Fig. 7B). Median fluorescence intensity values show that DHR123‐loaded HL‐60 neutrophils incubated with PMA have the highest fluorescence intensity after 1‐h incubation period (Fig. 7C).

**Figure 8:**
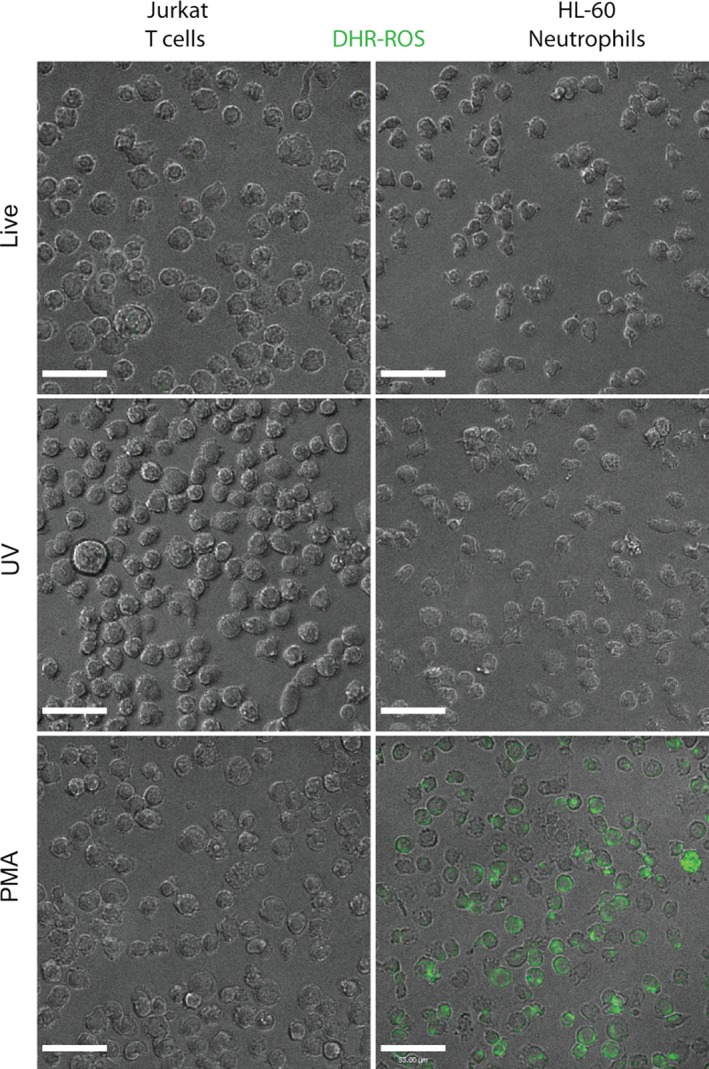
Fluorescence microscopy correctly detects the presence of ROS inside the cells. Jurkat T cells and HL‐60 neutrophils were treated with 25 nmol/L PMA or irradiated with UV light. Fluorescence microscopy of the cells 1 h after incubation with 25 *μ*mol/L DHR123 shows that HL‐60 neutrophils treated with PMA generate a large amount of ROS compared to other conditions. *N* = 3. Experiments were conducted in three different days; each sample was analyzed in duplicate chamber slides. Representative images are shown. DHR123 is represented by green color. Scale bar^ ^= 50 *μ*m. ROS, reactive oxygen species.

Imaging these cells by fluorescence microscopy showed that incubation of Jurkat T cells and HL‐60 cells with DHR after UV irradiation, or without any stimulation for 1‐h resulted in the detection of very little signal (Fig. 8). By contrast, large numbers of HL‐60 neutrophils, but not Jurkat cells, show intense green signal after stimulating the DHR123‐loaded cells with PMA. These data indicate that fluorescent microscopy and flow cytometry are suitable tools to study ROS production inside the cells that produce large amounts of ROS using DHR123.

## Discussion

DHR123 is a common fluorescence probe used for analyzing the production of ROS in cells undergoing oxidative stress (Gomes et al. 2005). However, the effects of using DHR123 in experiments involving UV irradiation of a variety of cell types in various buffer conditions have not been clearly established. This study shows that the DHR123 concentration and the duration of UV irradiation each directly increase the intensity of DHR123 green fluorescence, and that DMSO further increases the green fluorescence in certain buffers and media. These are key points because typical subtraction of values for buffers and cells (without UV irradiation) would not account for the observed effect of UV on DRH123. A spike in green fluorescence signal was observed immediately after UV irradiation of DHR123‐loaded A549 epithelial cells, 3T3 fibroblasts, and T cells. This initial increase is attributable to the direct effect of UV irradiation on DHR123, but not de novo ROS production. However, DHR123 can be used in HL‐60 neutrophils and primary neutrophils to detect ROS production. In terms of techniques, flow cytometry and fluorescence microscopy are superior to plate reader assays for measuring ROS production. Therefore, using DHR123 for measuring ROS in UV‐based experiments could give false‐positive results, and require great care in selecting experimental approaches.

Data obtained from a cell‐free system clearly show that UV irradiation directly increases the green fluorescence of DHR123 in concentration‐ and duration of UV irradiation‐dependent manners (Fig. 1). This increase is attributable to UV light oxidizing DHR123 to R123, consistent with the data obtained from a previous study, which suggests that UV irradiation can increase DHR123 oxidation without the presence of any ROS (Boulton et al. 2011). The type of UV used for irradiation (e.g., UV‐B, UV‐C) could also differentially affect DHR123 fluorescence. UV‐B in particular has been shown to generate a higher green signal from DHR123 compared to UV‐C (Horikawa‐Miura et al. 2007). Higher fluorescence intensity of DHR123 detected in various buffers is attributable to the different types of pigments and buffer salts present in the media (Fig. 2) because some of these components could increase the oxidation of DHR123 (Gomes et al. 2005). DMSO is a reagent commonly used as a carrier of various drugs and compounds in both in vitro and in vivo experiments (Champelovier et al. 2013; Douda et al. 2014, 2015). The fluorescence intensity remains unchanged for RPMI and DMEM compared to PBS and HBSS in the presence of various DMSO concentrations (0–1.25%, v/v). A previous study showed that DMEM could increase green fluorescence of DHR123 (Cunningham et al. 1985) and that DMSO increases the fluorescence of DHR123 in the absence of ROS (Horikawa‐Miura et al. 2007). Redox‐active metals such as Fe^2+^, and Cytochrome C in mitochondria during UV‐induced apoptosis also oxidize DHR123 (Qian and Buettner 1999). Moreover, compounds such as riboflavin and some ions present in commonly used culture media can oxidize DHR123 and interfere with DHR123‐mediated detection of ROS. Furthermore, HOCl and other compounds, but not H_2_O_2_ itself can oxidize DHR123 (Crow 1997). Therefore, buffers have to be carefully chosen when studying ROS with DHR123 and UV irradiation.

It is a common practice to pre‐load DHR123 into cells and tissues, and UV irradiate them or treat them with PMA to monitor ROS production during different types of cell deaths (Douda et al. 2014, 2015; Poniedzialek et al. 2015). This study shows that UV irradiation of DHR123‐preloaded A549 epithelial cells, 3T3 fibroblasts, Jurkat T cells, or primary T cells results in a rapid increase in fluorescence intensity compared to control cells (Figs. 4, 5). Nevertheless, adding DHR123 after UV irradiation shows no such increase in fluorescence signal. Thus, DHR123 is not useful for measuring ROS in A549, 3T3, Jurkat T cells, and primary T cells in the context of cell death. DHR123 has been used in lymphocytes to measure ROS (Poniedzialek et al. 2015); however, the data presented here (Figs. 2, 3, 4) indicate that caution is needed in the use of DHR123 in such studies. Interpreting DHR123 results would be expected to be more complicated in cultures with mix cell populations. This is because different cells induce different degrees of ROS, and that soluble or pre‐loaded DHR123 present in the cultures will contribute to erroneous interpretations of the degree of ROS production, particularly when plate reader assays are used.

Although the use of DHR123 is problematic in many cells that produce relatively small amount of ROS via mitochondrial pathways during UV irradiation, neutrophils generate large amount of ROS when treated with an NADPH oxidase activator such as PMA (Douda et al. 2014, 2015). Similar to non‐neutrophil cells (A549 epithelial cells, 3T3 fibroblasts and T cells), neutrophils (HL‐60 and primary cells) also generate nondetectable ROS upon UV irradiation (Figs. 4, 5, 6). However, a large amount of ROS is detectable by the DHR123‐based method in HL‐60 neutrophils and primary neutrophils activated with PMA (Fig. 6). In fact, H_2_O_2_ does not directly react with DHR123 and it requires peroxidase to convert H_2_O_2_ into other intermediates that react with DHR123 and generate R123 (Gomes et al. 2005). Cell‐free experiments show that even though some of the DHR123 molecules were oxidized upon UV irradiation, the remaining dye molecules are sufficient for detecting ROS in cells such as neutrophils (Figs. 3, 6). The amount of DHR123 molecules remaining in human skin HaCaT keratinocytes after UV irradiation is reported to be not sufficient to react with ROS (Boulton et al. 2011). Therefore, DHR123 could only be used for measuring ROS in certain types of cells such as neutrophils with appropriate experimental conditions.

In addition to plate reader assays, flow cytometry and fluorescence microscopy have been used for measuring ROS production using DHR123. This study shows that flow cytometry and fluorescence microscopy provide reliable results (Figs. 7, 8). The use of multiple techniques provides additional assurance of the correct interpretation of ROS production data measured with DHR123 (Douda et al. 2014, 2015). Collectively, to correctly interpret the DHR123‐based ROS data, several factors such as buffers, media, DMSO concentration, UV irradiation, and the types of cells should be taken into consideration.

## Authorship Contributions

Participated in research design: Djiadeu, Azzouz, Palaniyar; Conducted experiments: Djiadeu, Azzouz, Khan; Performed data analysis: Djiadeu, Azzouz, Khan, Palaniyar; Wrote or contributed to the writing of the manuscript: Djiadeu, Azzouz, Kotra, Sweezey, Palaniyar; Principal investigator and supervisor of the study: Palaniyar.

## Disclosure

None declared.
